# Looking into the Skin in Health and Disease: From Microscopy Imaging Techniques to Molecular Analysis

**DOI:** 10.3390/ijms241813737

**Published:** 2023-09-06

**Authors:** Constantin Caruntu, Mihaela Adriana Ilie, Monica Neagu

**Affiliations:** 1 Department of Physiology, The “Carol Davila” University of Medicine and Pharmacy, 050474 Bucharest, Romania; costin.caruntu@gmail.com; 2 Department of Dermatology, “Prof. N.C. Paulescu” National Institute of Diabetes, Nutrition and Metabolic Diseases, 011233 Bucharest, Romania; 3 Dermatology Department, Kalmar County Hospital, 391 85 Kalmar, Sweden; 4 Faculty of Biology, University of Bucharest, Splaiul Independentei 91-95, 050095 Bucharest, Romania; neagu.monica@gmail.com; 5 Immunology Department, “Victor Babes” National Institute of Pathology, 050096 Bucharest, Romania; 6 Department of Pathology, Colentina University Hospital, 020125 Bucharest, Romania

The skin is a complex organ that includes a wide variety of tissue types with different embryological origins. It is structured in different layers that are functionally interdependent. On the one hand, it plays a protective role against potentially aggressive environmental factors; on the other hand, it is also a communication interface between the body and the environment [1,2]. The skin undergoes a constant process of adaptation to various physiological and pathological conditions. Processes such as the maintenance of the integrity of the skin barrier, cutaneous regeneration, skin aging, skin inflammation, and carcinogenesis are currently topics of major interest for the research and medical communities [2,3]. The aim of our Special Issue was to expand our understanding and to emphasize new research directions related to the investigation of various aspects of the physiology and pathology of the skin, with a special focus on cellular and molecular mechanisms.

Skin cancer is the most common type of malignancy worldwide, and comprises two major types of cancer—melanoma, which is derived from the pigmentary cells of the skin, and non-melanoma skin cancer, developed from keratinocytes or their precursors—together with other, less common types of tumors [4,5,6,7,8,9,10].

Actinic keratosis (AK) is a very common premalignant skin lesion, which has the potential to progress to keratinocyte carcinoma [11]. Its high incidence and prevalence, as well as the high recurrence rate after treatment, makes AK a major problem for the public health systems [12,13]. However, the clinical or pathological markers for the progressive character of the lesion are still missing.

In our Special Issue, the research of Dubois-Pot-Schneider et al. [14] evaluates a transcriptomic approach to identify biological features, allowing them to objectively differentiate distinct AK subclasses. The study, which has the advantage of being performed on a large number of lesions, proposed a risk stratification of AKs based on their specific transcriptomic profile for the first time. The authors have described two different AK signatures. One resembles normal skin, and was defined as the lower risk, non-lesional type. The other, with a molecular profile similar to malignant lesions, is defined as the lesional type of AK, and carries a higher risk of evolving into cancer. The results indicate that, in the high-risk Aks, the upregulated genes are connected with inflammation, and the downregulated ones are related to the process of keratinization. These data suggest a very similar pattern to skin squamous cell carcinoma [15,16,17]. Moreover, they identified the VEGF pathway as being involved in the high-risk lesions, suggesting it as a potential therapeutic target [18,19].

Melanoma is an aggressive skin malignancy with a rapidly increasing incidence and high mortality rate, inducing an important impact on healthcare systems [20,21,22]. The complex melanoma pathogenesis involves various risk factors, including genetic susceptibility to UV exposure, chronic inflammation, and impaired immune responses [23,24,25,26,27,28]. If diagnosed early, melanoma is curable; however, in advanced stages, it involves complex and costly therapeutic strategies [20]. Thus, more and more studies have focused on the development of new strategies for detection of melanomas in the early stages. In recent years, the development of artificial intelligence and machine learning capabilities have opened new areas of research in the diagnosis of melanomas [29]. In our Special Issue, Foahom Gouabou et al. show the design of a new framework for automated melanoma diagnosis [30], which is easier to decrypt as regards the decision process; this automated diagnosis shows an increased performance as compared to previous systems.

Continuing the topic of our Special Issue, Dobre et al. emphasized the importance of early diagnosis in skin cancer once again, reviewing the most recent and relevant discoveries in the field of imaging techniques used for the diagnosis and therapeutic monitoring of skin cancer [31]. The implementation of non-invasive anatomical imaging techniques, such as confocal laser scanning microscopy (CLSM), optical coherence tomography (OCT), multiphoton microscopy (MPM), high-frequency ultrasound (HFUS), terahertz pulsed imaging (TPI), and magnetic resonance imaging (MRI), has provided better sensitivity and specificity, increasing the diagnostic reliability in skin cancer and premalignant lesions. For example, CLSM is one of the most promising techniques for micromorphological investigation in dermato-oncology, allowing examination of skin structures with a resolution comparable to that of conventional histopathology. It has been shown that CLSM is able to identify key features in different types of skin malignancies or premalignant lesions, being a performant, non-invasive diagnostic tool [32,33,34,35,36,37,38,39,40] with increased accuracy as compared to previous non-invasive approaches [41,42,43,44,45,46]. Moreover, it is helpful for identification of distinct tumor subtypes with specific malignant behavior [47,48,49]; CLSM can also be used for the evaluation of tumor edges and surgical margins [50,51], and offers the advantage of non-invasive monitoring of the therapeutic response [52,53,54,55,56,57,58,59,60]. Furthermore, the major advances in molecular imaging techniques, such as single photon emission computed tomography (SPECT/CT) and positron emission tomography (PET), and the recent burst in artificial intelligence research, have expanded the boundaries for the investigation of skin cancer, and are proven to be valuable tools for its detection and monitoring [61,62,63].

In the non-invasive imaging techniques domain highlighted by our Special Issue, the study by Tianxin Gao et al. [64] also focused on non-invasive imaging techniques, proposing a segmentation algorithm based on a deep learning network architecture for the segmentation of OCT images of laser-induced skin damage. The authors used an experimental model on adult BALB/c-mu mice, in which damaged skin areas with various degrees of injury were generated using different radiation doses emitted by a laser source. The skin injuries were investigated using an OCT system, and a deep neural network method was used to achieve accurate segmentation of the OCT images. The evaluation has produced good results, with a high overlap rate and short edge distance between the segmentation of OCT images and the manually labeled areas. These results suggest the possibility of using automated processing methods in the rapid detection and monitoring of skin lesions and the healing process.

The skin regeneration process was another topic explored by Yaotao Guo et al. [65]. They reviewed the fundamental mechanisms associated with skin soft tissue expansion and the involvement of the mechanical stretch process. Skin soft tissue expansion is a common technique in reconstructive specialties, such as plastic surgery and oral and maxillofacial surgery, being widely used in various conditions [66,67].

The action of the mechanical stretch on the skin activates multiple signaling pathways, inducing the activation of cell proliferation, differentiation, and migration. These activation processes involve all layers of the skin, inducing a shift in the behavior of keratinocytes, fibroblasts, and mesenchymal stem cells. Moreover, changes also occur in the hypodermis, blood vessels, and skin annexes [68,69]. By modulation of these pathways through manipulation of signaling molecules, and increased local supply of growth factors or active cells, the process of skin regeneration associated with soft tissue expansion can be improved.

Skin is constantly exposed to different environmental factors, which may activate potentially harmful processes. In our Special Issue, the effects of tris (1-chloro-2-propyl) phosphate (TCPP)—one of the most used organophosphorus flame retardants [70]—on human skin cells was investigated by Liu at al. [71]. In an in vitro research model on human skin keratinocytes (HaCaT), the authors have shown that TCPP exposure generates intracellular reactive oxygen species, triggers DNA damage, and disturbs the cell cycle control. Moreover, it increases the level of proinflammatory cytokines IL-1beta and IL-6. The keratinocytes’ viability is reduced in a concentration-dependent manner, and activation of the pathways involved in cellular senescence suggests that TCPP exposure can be a precipitating factor for skin aging.

Various intrinsic and environmental factors may be involved in skin aging, which is a process of fascinating complexity [72,73]. On the other hand, in recent years, the interest in regenerative medicine has been increasing, and numerous efforts have been made for the development of various anti-aging strategies [74,75].

Adipose-derived stem cells (ASCs) are multipotent cells with a high proliferation ability, which also have important regulatory functions [76]. Their role in rejuvenation and wound healing has been investigated, and the results are promising. ASCs are able to liberate various growth factors, to stimulate secretion of collagen and elastin, and to promote angiogenesis [76,77,78,79,80]. Therefore, the experimental study by Oh et al. published in this Special Issue investigated the impact of high-intensity focused ultrasound (HIFU) on ASCs and adipogenesis [81]. Their results indicate that HIFU modulates the functioning of ASCs by increasing the expression of the heat shock proteins 70 and reducing proinflammatory cytokines, such as NF-κB, IL-6, and TNF-α. Moreover, HIFU intensifies the expression of adipogenesis markers, induces a higher number of adipocytes, and increases the thickness of subcutaneous adipose tissue, suggesting its possible role in rejuvenation procedures.

The skin barrier, along with the other epithelial linings, protects our body against aggressive factors from the environment, and also prevents the loss of essential molecules [82,83,84,85,86]. Its integrity is essential for maintaining the cutaneous and whole-body homeostasis. Stratum corneum plays a fundamental role in the barrier function of the skin, which depends critically on its molecular architecture [1,87,88,89,90]. For example, significant alterations in the structure of the stratum corneum and in its lipid composition were identified in skin conditions with an impaired barrier function, such as atopic dermatitis [91,92,93,94,95,96,97,98,99].

In the study published herein, Sjövall et al. [100] have demonstrated the possibility of determining the molecular composition of superficial layers in stratum corneum, and the spatial distribution of specific lipids using three-dimensional time-of-flight secondary ion mass spectrometry. Thus, this technique could be used to expand the knowledge regarding the skin barrier, and to evaluate the effects of different active pharmaceutical ingredients, cosmetic molecules, or penetration enhancers in relation to this cutaneous function.

Summing up the presented Special Issue, prodigious amounts of research have been carried out in the field of skin imaging using cellular and molecular biology techniques. This technological armamentarium has recently led to massive progress in the diagnosis, targeted treatment, and investigation of pathophysiology of skin cancer.

Nevertheless, new discoveries are in the research pipeline, and many more are needed, as there are still patients that develop skin diseases with no clear pathogenesis, nor an effective treatment.
